# Characterization of Bioactive Compounds, Mineral Content, and Antioxidant Activity in Bean Varieties Grown with Traditional Methods in Oaxaca, Mexico

**DOI:** 10.3390/antiox8010026

**Published:** 2019-01-16

**Authors:** Karen Vanesa Armendáriz-Fernández, Ibeth Marlene Herrera-Hernández, Ezequiel Muñoz-Márquez, Esteban Sánchez

**Affiliations:** 1 Universidad Tecnológica de Camargo, Campus Meoqui, Avenida Ignacio Zaragoza y Manuel Ojinaga. Cd. Meoqui, Chihuahua C.P 33130, Mexico; karenvane150@gmail.com (K.V.A.-F.); marleneherrera.ih@gmail.com (I.M.H.-H.); 2 Centro de Investigación en Alimentación y Desarrollo A. C., Avenida Cuarta Sur No. 3820 Fraccionamiento Vencedores del desierto. Delicias, Chihuahua C.P 33089, Mexico; emunoz@ciad.mx

**Keywords:** *Phaseolus vulgaris* L., biofortification, bioactive compounds, antioxidant capacity, human health

## Abstract

The aim of the current study is to characterize the bean varieties produced in the State of Oaxaca (Mexico) with the purpose of selecting varieties with the potential of being biofortified with micronutrients. Eleven bean varieties representative of the State of Oaxaca (Mexico) were harvested, the color was characterized and the physicochemical analysis, mineral content, antioxidant capacity, reducing power, and bioactive compounds were determined. Data obtained were subjected to a variance analysis for the difference between the means of the bean varieties studied through the 95% Tukey test. The results obtained show the significant difference was found among the 11 bean varieties harvested in Oaxaca (México). The analysis found five outstanding varieties with a larger quantity of favorable characteristics on the iron, zinc, protein content, antioxidant capacity and reducing power. The outstanding varieties relating Fe and Zn content were Michigan for Fe 67.4 ppm, Sangre de Toro 62.4 ppm, which showed the larger content of antioxidant capacity (82.12%) scavenging activity and Biche for Zn 34.7 ppm. The variety with a larger quantity of protein (26.66%) was Biche bean, followed by the Peruano bean with 24.91% of protein. The variety with larger content of reducing power 0.16% was Blanco Michigan bean. Lastly, it is also remarkable that such outstanding bean varieties are important to include in a biofortification program with micro-nutrients to improve the food safety and the nutrition in vulnerable communities of the urban and rural sector of Oaxaca (Mexico).

## 1. Introduction

Currently, malnutrition affects more than one billion people worldwide and is one of the most important problems seriously threatening human well-being. Its main triggers are poor nutrition and deficiencies regarding nutrients, vitamins, and proteins in basic everyday meals from urban, rural, and marginal sectors of the population. Diverse research has proven that malnutrition in the early stages of life may limit long-term learning and intellectual development, as well as promote the early onset of diseases such as obesity, cancer, hypertension, and diabetes [1,2].

In the State of Oaxaca, located in the South of Mexico, 69.5% of the population lives in poverty and social inequality. From this percentage of the population, it is in rural communities where the most serious cases of malnutrition exist. National institutions have calculated that more than 17,000 children are malnourished. The main causes, which have triggered the increase in these indexes, include the limited access to nutritious foods with high nutraceutical quality. These limitations are added to factors related to excess weight and obesity, specifically in urban settings, because of an excessive consumption of calories and low energetic consumption [3].

Common bean (*Phaseolus vulgaris* L.) is the most important legume for human consumption since it has a great nutrimental contribution in protein, carbohydrates, lipids, iron, calcium, B complex vitamins, and minerals [4]. Bean is considered a main food in the basic consumer goods in Mexico. At the same time, it is included in the diet of many people. This harvest is considered a highly functional food since it has great content of bioactive substances with a beneficial potential for the health, such as polyphenols, flavonoids, anthocyanins, among others, which contribute in a synergistic way with their therapeutic properties and may have a positive effect against some pathologies. They also serve as an excellent source of natural antioxidants for disease prevention and health promotion [5]. 

The presence of antioxidant compounds in foods consumed every day is extremely important for the health of human beings. Its importance consists in providing an increasing body defense mechanism against daily physical exhaustion and exhaustion originated from the resistance workout. They are also capable of reducing oxidative damage, which is believed to cause many diseases such as cancer, atherosclerosis, cardiovascular diseases, diabetes, cataracts, arthritis, and diseases related to immunodeficiency and aging [6].

In the case of diabetes, the possible mechanism of the antioxidants is related to the retardation of digestion in the carbohydrates, especially, glucose, from which its release is also regulated by the liver. Relating cancer, it is associated with the human genotypes related to the oxidative stress [7].

Overall, there is limited information about the characterization of bioactive compounds, antioxidant capacity, and mineral content present in a different variety of beans in Mexico, due to the great agrobiodiversity. As such, the aim of the current study was to characterize the mineral content, bioactive compounds, and their antioxidant activity in bean varieties produced in the State of Oaxaca (México) with the purpose of selecting varieties that have a potential of being bio-fortified with micronutrients. This leads to improvements in the food safety and nutrition in vulnerable communities of the urban and rural sector of Oaxaca (México).

## 2. Materials and Methods

### 2.1. Material

Eleven varieties of bean representatives of the State of Oaxaca (Mexico) were harvested (Table 1).

### 2.2. Preparation of the Sample

One hundred seeds of each variety were taken, which were subjected to a milling through small blender containers to obtain a fine flour. Such samples were used for the interest analysis. Table 2 shows the agronomic characteristics of the evaluated bean varieties.

### 2.3. Plant Analysis

#### 2.3.1. Color Analysis

For the color measure, the methods proposed by Aguirre-Santos y Goméz-Aldapa [8] were used. Several seeds of each variety were taken and placed in glass Petri dishes, where L, a and b parameters were measured using a Chroma Meter CR-400/410 equipment. The L parameter represents the luminosity, ranged from 0 (black) to 100 (white). “a” parameter may have positive (red) or negative (green) values. “b” parameter may have positive (yellow) or negative (blue) values. Measures were performed with three repetitions in order to obtain the averages. Chroma (C) and Hue (H) parameters were calculated with the L, a and b values. Chroma (C) refers to the color saturation, whereas (H) refers to the color shade. The bean color will be provided based on the parameters function: L* (luminosity), C* (chroma), H* (shade), in this order. 

#### 2.3.2. Physicochemical Analysis

##### Ash Determination

Ash determination was performed using the method proposed by the Mexican Standard NMX-F-066-S-1978 [13]. In a crucible to constant weight, a 1 g sample was weighed with two repetitions for each variety, they were placed in the desiccator, and then the crucible was placed with samples in a muffle (Felisa) at 600 °C, to carbonize the sample until reaching the calcination. The results obtained from the ash were expressed as a percentage.

##### Fat Determination

The fat determination in the bean samples was carried out under the method Goldfish proposed by the Association of Official Analytical Chemists [14]. Goldfish Flasks were prepared to dry them in the stove until reaching the constant weight. Then Goldfish fat extractor LABCONCO^®^ equipment was mounted and the sample was placed inside filter papers and was covered with cotton, to be introduced inside the equipment. The solvent (petroleum ether) was added and was kept in reflux for 2.5 h. After the end of the extraction, the solvent was recovered by means of a distillation, retaining only the fat in the flask. Lastly, the flask with the waste was weighed and the fat percentage was determined. 

##### Moisture Content

Humidity determination was performed using the drying method in the open capsule proposed by the Association of Official Analytical Chemists [15]. For this analysis it was necessary to take 1 g sample for each repetition, with two repetitions for each bean variety. This was weighed in an aluminum capsule and previously dried at 75 °C until constant weight. After weighing each capsule, they were placed in the oven (Felisa^®^) for 12 h at 75 °C. Then, the capsules were removed from the oven and placed in the desiccator. Subsequently, they were reweighted. The humidity determination was expressed as a percentage. 

##### Fiber Determination

For the determination of the crude fiber, the method proposed by the Mexican Standard NMX-F-90-S-1978 [16] was used. From the sample that was previously degreased, this determination was performed. The samples were weighted, and their weights were registered. Each sample was transferred to the glass for fiber and then each glass was added with 200 mL 1.25% sulfuric acid and with 1 mL of isoamyl alcohol as antifoaming. The mixture was left for 30 min boiling. Lastly the rinses were performed to eliminate the waste of sulfuric acid and the isoamyl alcohol and at the same time to neutralize the mixture. Subsequently, a sample of 200 mL 1.25 sulfuric acid was added and was left boiling for 30 min and was then rinsed in fiberglass until neutrality were performed. Subsequently, fiberglass with the sample in the capsule was placed and put in the stove and allowed to dry for 12 h to make sure that the sample was completely dried. Once the drying is concluded, the capsule was weighted with the fiberglass and the sample, by the difference of weights the percentage of fiber contained in each sample was determined.

##### Carbohydrates Determination

Carbohydrates determination was performed by the difference of the other parameters and the percentage was reported [15].

##### Protein Determination

The protein determination by the Dumas method proposed by Calvo et al. [17] was used. First, a sample capsule with 3 µg nickel was taken and added 9 µg of vanadium pentoxide. Then, it was placed in the Flash 2000 (Thermo Scientific^®^ Corporation, Cambridge, UK) equipment, and the protein concentration was expressed as a percentage.

##### Energy Determination

The energy contained on each of the samples was measured by the sum of calories contained in carbohydrates, fat, and proteins in accordance with what is described by the Official Mexican Norm NOM-051-SCFI/SSA1-2010 [18]. The energy was expressed in Kcal/100 g. 

#### 2.3.3. Mineral Analysis

##### Micronutrients Determination

A 1 g sample was weighted in the analytical balance. Then, the digestion of each of the samples was performed. For that, a mixture with 100 mL nitric acid and 25 mL sulfuric acid (triacid mixture) was prepared, and for each gram of ground sample 25 mL of triacid mixture was added. The sample along with the acid was poured in 250 mL beakers and they were placed on a digestor rack (LABCONCO^®^ Corporation, Kansas City, MO, USA). For each beaker, a watch glass was placed with 3 boiling beads while the sample was digested in the rack. Once the sample was digested with the acid, it was poured in 50 mL flasks with glass funnels and paper filter (with the purpose of obtaining the purest sample). Then the flasks were graduated with tri-distilled water and were poured in 50 mL calibrated tubes for centrifuge. Fe, Zn, Mn, Cu, and Ni concentration was determined by Atomic absorption spectrophotometry (atomic absorption spectrophotometer iCE 3000 Thermo Scientific^®^) and was expressed in ppm for micronutrients.

##### Macronutrients Determination

Atomic absorption spectrophotometry (atomic absorption spectrophotometer iCE 3000 Thermo Scientific^®^ Corporation, Cambridge, UK) determined Mg, K, Ca, and the concentration in the same way the micronutrients were determined, and it was expressed as a percentage for macronutrients: K, Ca, Mg and Na. Phosphorus (P) determination was performed by the method of ammonium metavanadate in a range of absorption of 430 nm facing a calibration curve of K_2_HPO_4_. For the preparation of the phosphorous reagent, a beaker with 800 mL of hot deionized H_2_O, almost boiling, was used. Additionally, 10 g of ammonium molybdate and 0.5 g of vanadate-ammonium were dissolved. Continually, when it was already cold, 4 mL of HNO_3_ was added drop-by-drop at the beginning and then was continuously stirred. Subsequently, 134 mL of HNO_3_ was added. It was graduated with deionized H_2_O until reaching a final volume of 1 L. Then, 3.5 mL of tri-distilled water was added in test tubes (2 tubes per each sample repetition) and 500 µL of the sample of the previously digested variety was added. Finally, 1 mL of phosphorous reagent was added. Each tube was passed by a vortex and stood for one hour. At the end of the hour, the reading of each sample in the visible light spectrophotometer equipment (JENWAY Spectrophotometer, Jenway Limited^®^, Essex, England) was performed. The P concentration is expressed as a percentage of dried weight.

#### 2.3.4. Antioxidant Capacity Determination (2,2-diphenyl-1-picrylhydrazyl (DPPH)) 

The analysis was carried out with the method proposed by Hsu et al. [19]. The extract was obtained by macerating 1 g of seed in 5 mL of 80% methanol. Then it was centrifuged at 6000 rpm for 10 min. From the resulting supernatant 0.5 mL of the extract was taken and mixed with 2.5 mL of a freshly prepared DPPH 0.1 mM solution. Then, the mixture was incubated for 60 s in dark and cold conditions. The antioxidant capacity results were reported in the percentage scavenging activity.

#### 2.3.5. Reducing Power Determination

The reducing power in the seeds was measured according to the method described by Hsu et al. [19]. In addition, 1 g of the sample was macerated in ice with 5 mL of 80% methanol. It was subjected to a 500 rpm centrifugation for 10 min, from the resulting supernatant 1 mL of the extract was taken and then was added: 1 mL of 0.2 M phosphate buffer pH 6.6 and 1 mL of 1% K_3_Fe(CN)_6_ (J.T. Baker, Estado de Mexico, Mexico) (weight/volume). Subsequently, the mixture was incubated for 20 min at 50 °C. Then, the tubes were cooled and immersed for 10 min on ice and 0.5 mL of 10% Cl_3_CCOOH (Sigma-Aldrich, St. Louis, MO, USA) was added. After 10 min, they were centrifuged at 5000 rpm for 10 min while 1 mL of the resulting supernatant was taken and mixed with 1 mL of distilled water and 0.1 mL of FeCl_3_ (0.1%) (J.T. Baker, Estado de Mexico, Mexico). The mixture was incubated for 10 min at room temperature in a dark condition. The highest absorbance values show a higher reducing power. 

#### 2.3.6. Bioactive Compounds

##### Extraction and Quantification of Total Phenols

The extraction of phenolic compounds was determined with the colorimetry method facing a calibration curve of caffeic acid (10–100 μg/mL) proposed by Singlenton et al. [20]. A quantity of 0.5 g seeds was mixed with 2.5 mL of methanol, 2.5 mL of chloroform and 1.25 mL of NaCl (J.T. Baker, Estado de Mexico, Mexico) al 2% solution. Subsequently, it was homogenized. After a 5000 rpm centrifugation for 10 min, the following phases were obtained: methanolic phase, which has the phenolic acids (such a phase is used in the quantification process as extract). Dissolved lipids develop the interface, which is made by proteins precipitated by NaCl and the chloroform phase. Relating the phenol quantification, 750 µL of 2%, Na_2_CO_3_ (J.T. Baker, Estado de Mexico, Mexico) was added to the 50% 250 µL of 50% reagent Folin-Ciocalteau (Sigma-Aldrich, St. Louis, MO, USA) and 1375 µL of deionized H_2_O, 250 µL of enzymatic extract. Lastly, it was incubated at room temperature for 60 min. The results of total Phenol were expressed in mg of Caffeic acid g^−1^ (Sigma-Aldrich, St. Louis, MO, USA) (GA/g extract).

##### Flavonoids

Flavonoid analysis was performed with the method proposed by Zhishen et al. [21]. The extract was obtained macerating 0.5 g of ground seeds in 5 mL 80% methanol. Subsequently, it was centrifuged at 4000 rpm for 10 min. The reagent mixture consisted in placing 250 µL of the aliquot in the test tube, then adding 75 µL of NaNO_2_ (J.T. Baker, Estado de Mexico, Mexico), and it was passed by a vortex. It was allowed to rest for 5 min. After the 5 min, 150 µL of AlCl_3_ (Sigma-Aldrich, St. Louis, MO, USA) and 500 µL of NaOH (J.T. Baker, Estado de Mexico, Mexico) were added, dissolved with a final volume of 2.025 mL of H_2_O. The results obtained were expressed as equivalent of catechin per gram of sample (mg CE/g) based on dry weight.

##### Monomeric Anthocyanins

To determine the monomeric anthocyanins content, a pH differential method proposed by Wrolstad et al. [22] was used. A 0.5 g quantity was mixed with 5 mL methanol. Then, it was centrifuged at 4000 rpm for 10 min. After the centrifugation, two phases were obtained. From the first one, 0.5 mL was taken and 2 mL of potassium chloride was added, subjected to Vortex, then it was measured by spectrophotometry *A_460_*. For the second phase, 0.5 mL was taken and 2 mL of sodium acetate was added, subjected to Vortex. Then, it was measured by spectrophotometry. The results were reported as mg of cyanidin-3-glucoside (C3G)/g of the sample (dry weight).

#### 2.3.7. Statistical Analysis

All data were subjected to a variance analysis. For the difference between the bean varieties studied, the 95% Tukey test [23] was used. The significance levels of both analyses were expressed as: * *p* < 0.05, ** *p* < 0.01, *** *p* < 0.001, and NS (not significant).

## 3. Results and Discussion

This section may be divided by subheadings. It should provide a concise and precise description of the experimental results. Their interpretation as well as the experimental conclusions can be drawn.

### 3.1. Color Analysis

The color of the bean grain was determined by luminosity (L*), chromatic coordinates (a* and b*), the relative purity of the color (Croma), and Hue° angle, as shown in Table 3.

In relation to the current luminosity in such varieties, it is shown that the results range from 25.00% to 72.91% related to L*, which is the variety of bean Bayo (white color) including the one showing greater luminosity with 72.91%, whereas the variety of Negro bola shows the lower with 25.00% (Table 2). Indicating the luminous color present in these grains, since the color is darker, the luminosity is close to zero. That is why our varieties with the lighter shades in relation to the rest of varieties in the study, in the color of seed coat, had higher values related to luminosity, such as Blanco Michigan, Blanco Alubia and Bayo. Whereas the varieties with the lowest values are: Michigan, Negro Bola, and Sangre de Toro, due to its visible color in the seed coat are dark (Table 3).

In regard to chromatic coordinates a* and b*, the values for the samples in a* presented positive values which are from **0.93** for the Michigan variety up to **21.37** (*p* ≤ 0.001) for the Sangre de Toro variety. Taking into consideration the chromatic diagram of the L*a*b* space, it is observed that the value **0.93** tends towards the color green in the scale, while the value **21.37** tends towards the color red in the scale. The values for the samples in b* resulted in a negative value for the Michigan variety (0.61, *p* ≤ 0.001) and of **3.34** for Sangre de Toro. This indicates that such values tend towards the color blue of the scale. The CIE L*A*B* model is concurrent with the color of the Sangre de Toro seed coat since its values are within the red and blue scales. The shade is darker and is a darker red shade, which is characteristic of this variety. 

With a significance level of *p* ≤ 0.001 for b* values from the negative ones (−0.61) to the higher and positive one (25.47), it means that there is a significative difference in the variety of Michigan with **−0.61**, and higher than the Peruano variety with a value of **25.47** (Table 3). It is noted that within the chromatic model the negative values relating letter b* indicate the presence of blue shades, whereas, the same letter, but positive in this case, shows yellow shades. The results obtained in this study align since the variety showing negative values (Michigan −0.61) has a dark color on its seed coat.

Within the purity related to the color, known as chroma or color key, values are ranging from **2.13** to **25.63** (*p* ≤ 0.001), with the maximum value to the variety of Peruano bean with a value of **25.63**, and the value of 2.13 representing the minimum for the variety of Michigan (Table 3). This because values range from 0 to 100, where 0 indicates a low saturation showing gray, black or white shades or more discoloration. Whereas 100 indicates high saturation, which shows that the color is more intense. For that, this confirms the values obtained in the current study since the variety Michigan obtained a lower chroma value of 2.13 (*p* ≤ 0.001) which indicates that the dark color on the seed coat is not intense within the gray and black scale, whereas the variety of Peruano due to its seed coat color obtained a middle saturation.

With respect to the shade or hue (due to its English translation °Hue), once the °Hue was measured the negative and positive values were obtained, with the positive value corresponding to the Peruano variety, which showed a value of 1.45 °Hue, and the Michigan variety which showed a negative value of −0.37 °Hue (Table 3). It must be mentioned that such a study is found within these ranges. For that reason, the Hue grades are one of the remarkable properties or attributes in the property of a color, in which a stimulus may be described to be equal or different as green, red, yellow, and blue. For this parameter, there was a significative difference of *p* ≤ 0.01 among the different varieties.

Seed coat color does not only define and characterize bean variety, but it also offers protection against seed pathogens. Herrera-Pérez et al. [24] mention that tegument (seed coat) thickness and color serve as a protective coat against pathogen invasion. Studies also allowed to sustain that the coat in white seeds provides less protection to the embryo, compared to the protection provided to those seeds with colored tegument [25,26]

### 3.2. Physicochemical Analysis

Results obtained in the physicochemical analysis showed significative differences (*p* ≤ 0.001) between the different varieties of bean that were studied (Table 4), which include fiber, protein, fat, carbohydrates, energy, humidity, and ash concentration presented in the bean grain. 

From eleven varieties of Oaxaca bean relating the fiber concentration, the values ranged from 1.40% to 3.21%. The variety of Flor de Mayo is the most remarkable with a maximum concentration (3.21%), compared to the others and the Biche showing the minimum concentration (1.40%) (Table 4). In a similar study performed by Chávez-Mendoza and Sánchez [27] on varieties of beans from other regions, the levels of crude fiber from 1.77% to 2.77% were shown, with ranges within the ones in which the values obtained in this project are found. A similar study conducted by Aguirre-Santos and Gomez-Aldapa [8] showed that fiber concentration ranged from 1.35% to 2.77%, which indicates that the values we obtained were greater to those identified by said authors. 

In relation to the protein content, in our study, the values obtained are found within a range from 21.5% to 26.6%, which is the variety of bean Biche and the remarkable one for showing 26.6%. This is the higher one, whereas the lower one is for the variety of bean Bayo with 21.5%. It is worth noting the difference between the Biche variety and the common consumption varieties such as Peruano, Negro Bola, and Pinto Americano, whose values are differential of 6.4%, 7.9%, and 11.28% (Table 4). Ulloa et al. [28] point out that protein values in the bean are found between 14% and 33%. For that reason, these results are found within such a range. According to Ulloa et al. [28], the protein values seen in beans range from 14% to 33%. The varieties analyzed in our study lie within this range.

Values obtained from fat show that the most outstanding variety of bean was the bean Blanco Alubia, which obtained a maximum value of 0.68%, whereas the minimum value obtained was found in the Peruano one with 0.79% (Table 4). A similar study performed by Barampama and Simard [29] reported that the fat median values in the bean are found as 1.01%. For that, results obtained in our varieties are found within such a range.

In respect to the carbohydrate content, the values obtained show that, for the variety of bean Bayo, the higher content was obtained to be 57.08%, whereas, for Sangre de Toro, the minimum value was 55.2%. The Negro Bola variety also showed a high concentration of carbohydrates with 57.0%, which is just 0.15% below the Bayo variety. For others of the varieties of frequent consumption among the population such as Pinto Americano and Peruano, the difference shown when compared to the highest value was just 0.5% and 0.85% (Table 4). With regard to the investigations conducted by Campos-Vega [30] and Rodríguez-Miranda et al. [31], where carbohydrate concentrations ranged from 51.5% to 56.2%, it is worth noting that the bean varieties in our study significantly exceeded the values obtained by said authors.

Regarding the energetic content present in the analyzed varieties of bean, there was a high value of 340.5 Kcal belonging to the variety of bean Biche, which is followed by the Blanco Michigan variety with 335.6 Kcal. Other varieties with intermediate energy values such as Peruano, Pinto Americano and Negro Bola remained at 2.12%, 2.0%, and 1.94% below the Biche variety. It is also important to mention that the lowest energetic content was for the variety Negro Michigan with 322.0 Kcal (Table 4).

In the results of ash and humidity, significative differences of *p* ≤ 0.001 among the studied varieties were found (Table 4). In the ash parameter, the most remarkable variety of bean was Sangre de Toro with 5.09% and the lower one was the variety of bean Biche with 3.61%. For the humidity parameter, the bean variety with the higher percentage was Michigan with 14.51% compared to the other varieties, as well as Negro Bola containing 10.32% being the minimum value (Table 4). According to investigations conducted by Campos-Vega [30] and Rodríguez-Miranda et al. [31], the moisture percentage ranges from 8.0% up to 11.95%.

### 3.3. Mineral Analysis

#### 3.3.1. Micronutrients Analysis

The results obtained in the micronutrient analysis showed significative differences in the varieties of studied beans. Values obtained are shown in Table 5. In which the Cooper (*p* ≤ 0.01), Nickel (*p* ≤ 0.001), Manganese (*p* ≤ 0.001), Iron (*p* ≤ 0.05), and Zinc (*p* ≤ 0.001) concentrations contained in the eleven varieties of bean are shown.

Regarding the Copper concentration, the notable values ranged from 4.6 to 12.1 ppm, which is the variety of Peruano bean for containing the maximum concentration of 12.1 ppm, whereas the variety of Bayo bean shows the minimum concentration with 4.6 ppm (Table 5).

Nickel content is found ranging from 12.7 and 29.1 ppm, which emphasizes the variety of bean Pinto Americano with the higher content of 29.1 ppm, related to the variety of bean Blanco Alubia showing the lower content of 12.7 ppm (Table 5).

Regarding the Manganese concentration, the variety of bean Flor de Mayo is showing the maximum level with a value of 29.8 ppm, related to the minimum level obtained in the variety Michigan with a value of 18.5 ppm (Table 5).

Regarding Iron, values range between 45.3–67.4 ppm. The variety of bean Negro Michigan have a maximum concentration of 67.4 ppm, whereas the variety of bean Blanco Alubia shows a minimum concentration with 45.3 ppm (Table 5). According to Guzmán-Maldonado et al. [4], who conducted a study on Mexican bean varieties, their study revealed that the iron concentration in beans ranged from 24.8 ppm to 57.5 ppm. These values are found over the ranges of a similar study performed by Reference [29] (42.3 ppm), which indicates that our values are higher due to their variety and region. It is important to note that the variety with the lowest concentration is Blanco Alubia with 45.3 ppm, and even so, it is found over the results of such a study.

On the other hand, values obtained for Zinc, are found ranging from 10.7 to 34.7 ppm. Additionally, the variety of bean Biche shows a higher concentration with 34.7 ppm. For that, a variety of bean Blanco Michigan shows the minimum concentration with 10.74 ppm (Table 5). These results are similar to those found by Guzmán-Maldonado et al. [4], who determined the zinc content found in a number of bean varieties from different parts of Mexico. According to Chávez-Mendoza and Sánchez [27] zinc values in varieties of beans from different regions of Mexico have concentrations of 30.8 ppm. Therefore, our varieties are found within such a range. 

#### 3.3.2. Macronutrients Analysis

Results obtained from the macronutrients analysis showed significative differences for nitrogen (*p* ≤ 0.001) and phosphorous (*p* ≤ 0.001) in eleven varieties of bean that were studied, while, for potassium, magnesium, and calcium, no significative difference was found. Values obtained were shown in Table 6. 

Regarding the nitrogen concentration, values range from 3.45% to 4.26%, which is the variety of bean Biche the most remarkable showing the maximum concentration with 4.26%, whereas the variety of bean Bayo shows the minimum concentration with 3.45% (Table 6).

With respect to the phosphorous concentration, two varieties with the same value were found to have both Peruano and Sangre de Toro showing the maximum concentration with 0.17%, compared to the Michigan and Bayo Bola beans with a minimum concentration of 0.01% (Table 6). A similar study performed by Velasco-Gonzales et al. [32] showed that the Phosphorous levels are found in 0.85% and 1.29%, As such, our varieties are found within such range. Regarding values obtained for potassium, the variety of bean Michigan shows the higher content of 1.05%, related to the variety of Biche bean that shows the lowest concentration with 0.33% (Table 6). 

On the other hand, values obtained for magnesium are found to range from 0.03% to 0.11% in which two varieties were noted with the same concentration with Negro Bola and Bayo Bola beans showing the higher concentration with 0.11%, whereas varieties of Biche and Pinto Americano beans are both showing the lowest concentration with 0.03% (Table 6). According to Velasco-Gonzales et al. [32], which indicated that their magnesium levels range between 0.10% and 0.17%, denote that our values are found within such range.

Among the analyzed bean varieties related to the calcium concentration, it has been observed that the maximum concentration was shown in the variety of Biche bean with 1.39%, as well as the minimum concentration with 0.08% showed by a variety of Bayo bean (Table 6).

### 3.4. Antioxidant Activity

**Figure 1:**
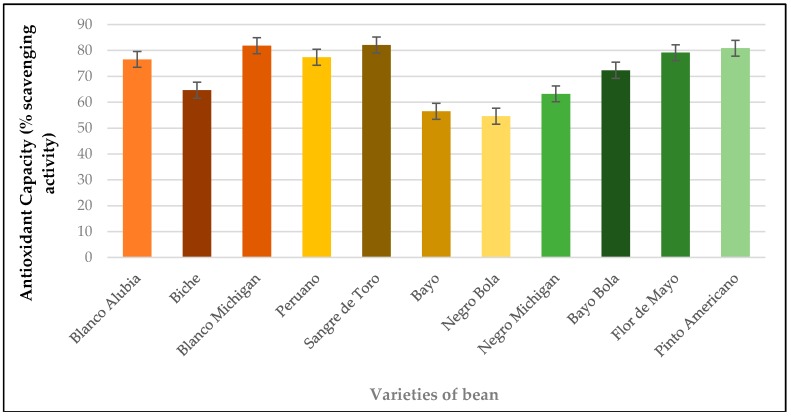
Antioxidant capacity in bean varieties produced in the State of Oaxaca. Data are the mean ± standard error (*n* = 3).

Values obtained for the antioxidant capacity in eleven varieties of bean from the State of Oaxaca, Mexico are shown in Figure 1. Eleven varieties of bean that were studied are classified into three main groups: (1) high levels, (2) medium levels, and (3) low levels of antioxidant capacity. Within the high level of antioxidant capacity are the following bean varieties: Sangre de Toro, Blanco Michigan, Pinto Americano, and Flor de Mayo. The Sangre de Toro variety has the highest antioxidant capacity (82.1%), followed by Blanco Michigan (81.8%), Pinto Americano (80.6%) and Flor de Mayo (79.1%). It is important to note the similarity between these varieties regarding the protein value, where the Sangre de Toro variety has 24.8%, followed closely by the Blanco Michigan (23.0%), Pinto Americano (23.0%), and Flor de Mayo (23.1%) varieties. In the middle level, the antioxidant capacity of the following bean varieties are found: Blanco Alubia, Biche, Peruano, Michigan, and Bayo Bola. Such varieties show a comparable color between brown and yellow except Michigan since it is black, which is the variety of Peruano bean the most remarkable among medium levels regarding its maximum concentration of antioxidant capacity with 77.3%. In the low level of antioxidant capacity, the following bean varieties are found: Bayo and Negro Bola, with the former showing the lowest level with 54.6%. From the eleven bean varieties, Sangre de Toro shows the maximum value of antioxidant capacity 82.1% compared with the variety of Negro Bola, which showed the minimum value of antioxidant capacity with 54.6%. This shows an increase of 33.5% related to the lowest variety of antioxidant capacity. 

In previous studies, carried out by Salinas [33] reports that the antioxidant capacity is related to compounds that give color to the seed coat and indicates that the color pink red and black in the seed coat are the carriers of the highest values of this variable. In our study, it is observed that the varieties with greater antioxidant activity are White Michigan, Blood of bull and American Pinto, whose colors of seed coat are clear and red, which indicates that aside from the color of the seed coat, influences the agronomic management of the crop and the environmental conditions in which they were developed.

### 3.5. Reducing Power

**Figure 2:**
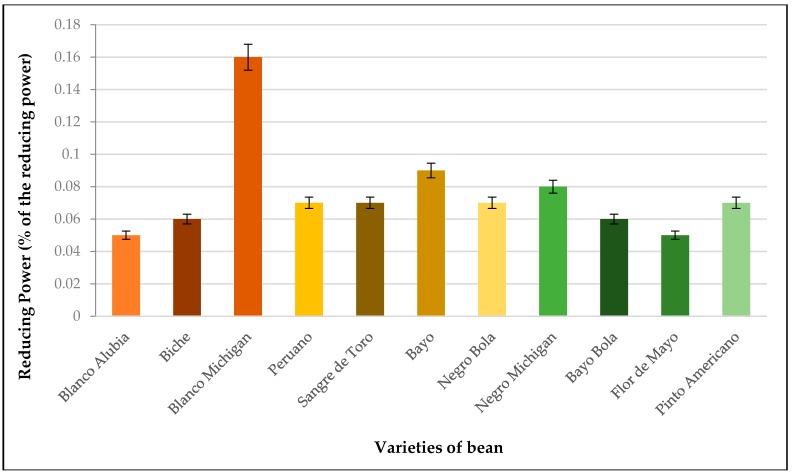
Reducing power in bean varieties produced in the State of Oaxaca. Data are means ± standard error (*n* = 3).

Values of reducing power in eleven varieties of bean produced in the State of Oaxaca (Mexico) are shown in Figure 2. These varieties of bean that were studied are classified into three main groups: (1) high levels, (2) medium levels, and (3) low levels of antioxidant capacity. Within the high level of antioxidant capacity, the following bean varieties are found: Blanco Michigan, Michigan, and Bayo are the variety of bean Blanco Michigan containing 0.16% of reducing power. The medium level of reducing power are found following analyzed varieties such as: Biche, Peruano, Sangre de Toro, Negro Bola, Bayo Bola, and Pinto Americano. However, the most remarkable varieties within this level is the Peruano, Sangre de Toro, Negro Bola, and Pinto Americano beans since such varieties contain 0.07% of reducing power, which shows, among them, 24% of protein. In the last level, the following bean varieties are found: Blanco Alubia and Flor de Mayo. Both varieties represent this level with 0.05% of reducing power. With the most remarkable among the eleven bean varieties, Blanco Michigan has the higher quantity of reducing power with 0.16% whereas the bean with lower percentage corresponds to the varieties of Blanco Alubia and Flor de Mayo both with 0.05% of reducing power. This shows an increase of 68.75% between the highest and lowest varieties. 

The reducing power of a compound can serve as an important indicator of its potential antioxidant activity [34]. In our study, the greater reducing power coincides with the greater antioxidant capacity of the bean, which could be explained by the higher content of phenolic compounds present in the bean varieties studied. These results coincide with Yildirim et al. [35], who determined the reducing power in leaves and seeds of *Rumex crispus* L. This confirms an increase according to the concentration of phenolic compounds, which presents a statistically significant correlation (*r* = 0.99). Thus, the reducing power of a compound can serve as an indicator of its antioxidant activity [35].

### 3.6. Bioactive Compounds

Values obtained from the bioactive compound analysis are shown in Table 7. The concentration of total phenols, flavonoids, and anthocyanins are presented in the bean grain. For the determination of total phenols (*p* ≤ 0.01) and anthocyanins (*p* ≤ 0.001), there is a significative difference among the species, whereas, for flavonoids, no significative difference was found. Relating the total phenol content present in bean varieties ranges from 58.98 to 109.54 mg Gallic Acid/g, with the Peruano the most remarkable having a higher content of 109.54 mg Gallic Acid/g, related to the variety Biche which shows a lower quantity with 58.98 mg Gallic Acid/g (Table 7). A previous study done by Gracia-Nava [36], who performed a quantification in total phenols, revealed results showing that phenol concentration ranges from 19.75 mg to 221.48 mg Gallic acid/g extract. These values match the value range observed in our study.

Regarding the flavonoid concentration, values range from l0.62 to 7.43 mg equivalent to Catechin per sample gram (mg CE/g), with the Pinto Americano variety holding the maximum concentration (7.43 mg CE/g), compared to Blanco Alubia showing the minimum concentration (0.62 mg CE/g) (Table 7). A similar study conducted by Gracia-Nava [36] reported a minimum value of 2.26 mg catechin/g extract, whereas the maximum value reported was 25.94 mg catechin/g extract. However, the values obtained in our study range from 0.62 to 7.43 mg catechin/g extract.

On the other hand, values obtained for anthocyanins, show that the most remarkable bean was Blanco Alubia with a maximum value of 2.33 mg/g of extract, whereas the minimum one was found in the variety Flor de Mayo with 0.39 mg/g of extract (Table 7). A study performed by Chávez-Mendoza and Sánchez [27] shows that its antioxidant levels were 0.43 mg/g of extract including a value in which the results obtained in this project are found. In a study conducted by Rocha-Guzmán et al. [37], levels of anthocyanins were reported to be 3.75 mg EC3G/g extract. When comparing his study to ours, we noticed that the bean variety shows the highest anthocyanins content in our study, which has a lower concentration even though such content is still considered satisfactory.

Seven variables in 11 varieties of bean from the State of Oaxaca (Mexico) were determined, of which five were individual and generally remarkable. Within the remarkable varieties the variety of bean Biche from the coastal region of Oaxaca (Mexico) is found, with higher content in Zinc (34.77 ppm) and protein (26.66%). Therefore, the Peruano variety from Sierra Norte of Oaxaca (Mexico), which is the second one with higher protein content (24.91%). Lastly, the Michigan variety is found on an individual basis, from the Central Valley of Oaxaca (México), which shows the higher content of iron (67.42 ppm). Generally, within the remarkable varieties, the bean Sangre de Toro from Sierra Norte of Oaxaca (Mexico) is found in the second one with the highest level of iron with (62.49 ppm). Moreover, it represents more compounds. Such a variety was the most outstanding in the antioxidant capacity with 82.12% scavenging activity due to its high content of flavonoids (4.24 mg CE/g) and anthocyanins (1.16 mg/g of extract). Lastly, there is a variety of Blanco Michigan from the coast region of Oaxaca (México), including more compounds in the second one, which has a high value in reducing power (0.16% of reducing power), due to the high content of phenols (98.77 mg Gallic acid/g), anthocyanins (2.03 mg/g extract), and antioxidant capacity (81.82% scavenging activity). Such remarkable varieties came from the same regions, in which they have some genetic variabilities due to the environment, soil, minerals, and weather, which has a mechanism to absorb more nutrients. 

## 4. Conclusions

From 11 varieties representative of the State of Oaxaca (Mexico) five of them were found remarkable to show higher values related to measured compounds. The variety of bean Biche showed the higher Zinc content (34.7 ppm) and protein (26.6%). Therefore, the Peruano variety is found and the second one has the highest content in protein with 24.9%. Relating the variety Negro Michigan, this showed the highest value in iron with 67.4 ppm. The variety of bean Sangre de Toro was the second one with the highest value in iron with (62.4 ppm) such a variety having the most remarkable related to antioxidant capacity with 82.12% scavenging activity due to its high content in flavonoids (4.24 mg CE/g) and anthocyanins (1.16 mg/g of extract). Lastly, the variety Blanco Michigan was found to show a high level in reducing power (0.16% of reducing power) due to its great content in phenols (98.77 mg Gallic acid/g), anthocyanins (2.03 mg/g extract) and antioxidant capacity (81.82% scavenging activity). The five outstanding bean varieties in the measured compounds are good prospects to be included in the biofortification program, and in this way improve nutrition and food security in vulnerable communities of the urban and rural sector of Oaxaca, Mexico.

## Figures and Tables

**Table 1:** Varieties of bean from the state of Oaxaca (Mexico).

Bean Variety	Origin	Date Obtained	Picture
Blanco Alubia	Yautepec, Oaxaca Región Sierra Sur	15 February 2018	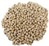
Biche	Juquila, Oaxaca Región Costa	15 February 2018	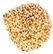
Blanco Michigan	Pochutla, Oaxaca Región Costa	15 February 2018	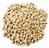
Peruano	Villa Alta, Oaxaca Sierra Norte	15 February 2018	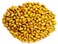
Sangre de Toro	Villa Alta, Oaxaca Sierra Norte	15 February 2018	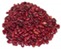
Bayo	Mixe, Oaxaca Sierra Norte	15 February 2018	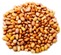
Negro Bola	Choapam, Oaxaca Región Papaloapan	15 February 2018	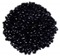
Negro Michigan	Zaacila, Oaxaca Valle Centrales	15 February 2018	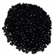
Bayo Bola	Tlacolula, Oaxaca Valle Centrales	15 February 2018	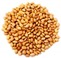
Flor de Mayo	Etla, Oaxaca Valle Centrales	15 February 2018	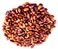
Pinto Americano	Centro, Oaxaca Valle Centrales	15 February 2018	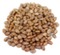

**Table 2:** Agronomic characteristics of evaluated bean varieties.

Bean Variety	Agronomic Characteristics
Blanco Alubia	Intermediate cycle with 50 days of flowering and 100 days until it reaches physiological maturity, tall crop (about 49 cm) with determined growth habit (Type I). Produces in average 14.8 bean pods per plant and 4.3 seeds per bean pod. Resistant to diseases such as rust and tolerant to the yellow mosaic virus (Bean Golden Mosaic Virus) and common mosaic virus (Bean Common Mosaic Virus) [9].
Biche	Short cycle (90 days), growth habit varies from erect variable with the undetermined guideline. Small beige to white seed, with a black halo surrounding the embryo [10].
Blanco Michigan	Undetermined growth habit, short guideline, erect (type IIa), intermediate vegetative cycle (45 days for flowering and 95 days to reach maturity), oval-shaped white seed, resistant to oxidation (color loss or aging) [11].
Peruano	Determined habit, erect, with branches close to the main stem (Type Ia), intermediate vegetative cycle (42 days for flowering and 94 days to reach maturity), light yellow seed (sulphur like yellow), without color around the hilum. Resistant to oxidation (loss of color or aging) due to storage period or due to rain during harvest. The seed is an elongated and oval shape, mid-size, weighing 35 g for 100 seeds. Resistant to rust and halo blight. With intermediate resistance to anthracnose, angular spot, and common bacterial diseases. Intermediate yield, between 1500 to 2300 kg/ha commercially [11].
Sangre de Toro	Bush growth variety, vegetative cycle from 70 to 90 days, elongated and round seed with central embryo scar, dark and shiny red seed. [11]
Bayo	Undetermined habit, prostrate, short guidelines (Type III a), the intermediate vegetative cycle of 42 days for flowering and 94 days to reach maturity. Intermediate to high yields, between 1500 to 2600 kg/ha. The seed is mid-sized, light-colored, with yellow hilum. Tolerant of oxidation (loss of color or aging) due to storage or rains during harvest. The approximate weight for 100 seeds is 30 g. Resistant to rust, halo blight, intermediate resistance to anthracnose, angular spot and common bacterial diseases [11].
Negro Bola	Undetermined type III growth habit, short guidelines, mid-sized. Its cycle is intermediate since it reaches maturity in 100 days, rich in anthocyanins. Small and round seed, resistant to rust, tolerant of anthracnose and bacterial diseases [12].
Negro Michigan	The plant is of undetermined growth habit, type II, short guides and erect, mid-size. Its cycle is intermediate and reaches maturity in 95 to 105 days. Semi-oval shaped seed, opaque, rich in anthocyanins [12].
Bayo Bola	Undetermined habit, prostrate, short guideline (type IIIa), intermediate vegetative cycle (44 days for flowering and 96 days to reach maturity). Resistant to rust and halo blight. Intermediate resistance to anthracnose, angular spot, and common bacterial diseases, small sized (100 seeds weigh 25 g) [11].
Flor de Mayo	Undetermined growth habit, prostrate and with the short guideline (type IIIa). Its vegetative cycle is intermediate with 44 days for flowering and 96 days to reach maturity. Its yield is from intermediate to high (1500 to 2600 kg/ha). The seed is pink-purple and beige with spots on the back. Prismatic, semi-flattened and mid-sized seed (100 seeds weigh 32 g). Resistant to rust and halo blight. Intermediate resistance to anthracnose, angular spot and common bacterial diseases [11].
Pinto Americano	The Pinto Americano bean crop cycle is from 80 to 85 days. Flowering is from 50 to 55 days. The habit of growth is indeterminate prostrate. The Pinto Americano variety is resistant to drought but with a high susceptibility to root rot associated with soil pathogens [11].

**Table 3:** Determination of colorimetry in bean varieties produced and consumed in the State of Oaxaca, Mexico.

Varieties of Bean	L*	a*	b*	Chroma	°Hue
Blanco Alubia	72.37	2.40	13.13	13.35	1.38
Biche	**72.91**	4.93	17.43	18.12	1.29
Blanco Michigan	71.66	2.47	12.61	12.84	1.37
Peruano	63.95	2.87	**25.47**	**25.63**	**1.45**
Sangre de Toro	30.41	**21.37**	3.34	21.63	0.15
Bayo	63.48	8.86	19.01	20.97	1.13
Negro Bola	25.00	2.22	0.35	2.47	0.02
Negro Michigan	25.07	0.93	−0.61	2.13	−0.37
Bayo Bola	60.43	9.08	23.76	25.45	1.20
Flor de Mayo	47.47	15.65	9.49	18.30	0.54
Pinto Americano	54.04	9.09	14.04	17.03	1.00
Significance	***	***	***	***	**
MSD	3.50	1.04	3.94	3.16	1.24

Significance level: * *p* ≤ 0.05, ** *p* ≤ 0.01, *** *p* ≤ 0.001, NS (without significance). NOTE: Numbers in bold represent the maximum values and the underlined numbers represent the minimum values.

**Table 4:** Physicochemical composition of bean varieties produced and consumed in the state of Oaxaca (Mexico).

Variety of Bean	Ash %	Fat %	Humidity %	Fiber %	Carbohydrate %	Protein %	Energy (Kcal)
Blanco Alubia	4.53 ± 0.03	1.68 ± 0.04	11.4 ± 0.03	2.44 ± 0.02	56.2 ± 0.11	23.7 ± 0.03	334.8 ± 0.01
Biche	3.61 ± 0.03	1.12 ± 0.02	11.2 ± 0.02	1.40 ± 0.03	55.9 ± 0.10	26.6 ± 0.03	340.5 ± 0.10
Blanco Michigan	4.37 ± 0.03	1.51 ± 0.03	11.1 ± 0.03	2.46 ± 0.02	56.8 ± 0.11	23.7 ± 0.03	335.6 ± 0.06
Peruano	4.64 ± 0.02	0.79 ± 0.03	10.6 ± 0.02	2.37 ± 0.03	56.6 ± 0.11	24.9 ± 0.03	333.3 ± 0.07
Sangre de Toro	5.09 ± 0.02	1.35 ± 0.02	11.0 ± 0.03	2.45 ± 0.02	55.2 ± 0.09	24.8 ± 0.03	332.5 ± 0.06
Bayo	4.44 ± 0.03	1.03 ± 0.03	13.0 ± 0.02	2.84 ± 0.02	57.08 ± 0.10	21.5 ± 0.03	323.9 ± 0.03
Negro Bola	4.60 ± 0.03	0.87 ± 0.03	10.3 ± 0.02	2.68 ± 0.09	57.00 ± 0.05	24.5 ± 0.03	333.9 ± 0.39
Negro Michigan	4.48 ± 0.02	1.62 ± 0.03	14.5 ± 0.02	2.52 ± 0.03	54.4 ± 0.09	22.4 ± 0.02	322.0 ± 0.06
Bayo Bola	4.58 ± 0.02	1.39 ± 0.02	12.9 ± 0.03	2.79 ± 0.02	56.0 ± 0.08	22.2 ± 0.02	325.7 ± 0.06
Flor de Mayo	4.51 ± 0.03	1.15 ± 0.02	12.8 ± 0.02	3.21 ± 0.03	55.0 ± 0.09	23.1 ± 0.02	323.3 ± 0.10
Pinto Americano	4.69 ± 0.02	1.33 ± 0.02	11.2 ± 0.02	2.31 ± 0.03	56.8 ± 0.09	23.6 ± 0.03	333.7 ± 0.11
Significance	***	***	***	***	***	***	***
MSD	0.011	0.020	0.009	0.010	0.100	0.011	0.414

Significance level: *** *p* ≤ 0.001, NS (no significance). NOTE: Numbers in bold represent the maximum values and the underlined numbers represent the minimum values.

**Table 5:** Micronutrients concentrations (ppm) in bean varieties produced and consumed in the State of Oaxaca, Mexico.

Variety of Bean	Copper	Nickel	Manganese	Iron	Zinc
Blanco Alubia (119)	7.4 ± 1.5	12.7 ± 1.6	21.3 ± 0.4	45.3 ± 3.7	33.0 ± 1.6
Biche (123)	9.5 ± 0.1	18.9 ± 0.6	20.5 ± 1.5	62.0 ± 9.7	**34.7 ± 3.2**
Blanco Michigan (124)	7.7 ± 2.0	18.0 ± 0.3	23.5 ± 1.1	57.2 ± 8.9	10.7 ± 0.4
Peruano (126)	**12.1 ± 1.5**	17.4 ± 0.2	19.1 ± 0.5	47.8 ± 2.4	26.4 ± 2.6
Sangre de Toro (127)	9.1 ± 0.7	21.1 ± 1.2	25.2 ± 1.2	62.4 ± 11.6	30.8 ± 1.8
Bayo (128)	4.6 ± 0.8	21.0 ± 0.3	23.2 ± 1.3	53.9 ± 1.0	17.2 ± 1.2
Negro Bola (129)	8.6 ± 0.09	18.5 ± 1.6	23.4 ± 2.1	50.9 ± 4.3	21.9 ± 2.3
Negro Michigan (134)	7.3 ± 1.3	25.1 ± 1.5	18.5 ± 0.8	**67.4 ± 7.3**	25.8 ± 2.9
Bayo Bola (135)	7.0 ± 0.3	27.1 ± 1.1	25.3 ± 3.08	54.3 ± 1.5	28.6 ± 6.5
Flor de Mayo (137)	5.9 ± 2.7	23.2 ± 0.7	**29.8 ± 2.1**	57.3 ± 4.5	23.9 ± 1.03
Pinto Americano (140)	7.6 ± 0.08	**29.1 ± 0.8**	26.1 ± 2.6	58.4 ± 1.2	23.4 ± 3.6
Significance	**	***	***	*	***
MSD	3.9	3.3	4.3	18.7	9.0

Significance level: * *p* ≤ 0.05, ** *p* ≤ 0.01, *** *p* ≤ 0.001, NS (no significance). NOTE: Numbers in bold represent the maximum values and the underlined numbers represent the minimum values.

**Table 6:** Macronutrient Concentrations (%) in bean varieties produced and consumed in the State of Oaxaca.

Varieties of Bean	Nitrogen	Phosphorous	Potassium	Magnesium	Calcium
Blanco Alubia (119)	3.7 ± 0.004	0.07 ± 0.020	0.42 ± 0.06	0.08 ± 0.07	0.53 ± 0.50
Biche (123)	**4.2 ± 0.004**	0.05 ± 0.004	0.33 ± 0.01	0.03 ± 0.02	**1.39 ± 1.21**
Blanco Michigan (124)	3.7 ± 0.004	0.12 ± 0.050	0.54 ± 0.08	0.07 ± 0.02	0.09 ± 0.07
Peruano (126)	3.9 ± 0.004	**0.17 ± 0.001**	0.80 ± 0.67	0.07 ± 0.06	0.28 ± 0.06
Sangre de Toro (127)	3.9 ± 0.004	**0.17 ± 0.003**	0.45 ± 0.02	0.04 ± 0.01	1.27 ± 0.63
Bayo (128)	3.4 ± 0.004	0.14 ± 0.020	0.47 ± 0.03	0.06 ± 0.02	0.08 ± 0.06
Negro Bola (129)	3.9 ± 0.004	0.15 ± 0.010	0.68 ± 0.12	0.11 ± 0.01	0.29 ± 0.06
Negro Michigan (134)	3.5 ± 0.003	0.01 ± 0.001	**1.05 ± 0.36**	0.07 ± 0.03	0.77 ± 0.79
Bayo Bola (135)	3.5 ± 0.003	0.01 ± 0.001	0.60 ± 0.25	**0.11 ± 0.01**	1.34 ± 2.09
Flor de Mayo (137)	3.7 ± 0.003	0.16 ± 0.010	0.41 ± 0.02	0.05 ± 0.02	0.26 ± 0.04
Pinto Americano (140)	3.7 ± 0.004	0.16 ± 0.010	0.42 ± 0.07	0.03 ± 0.02	0.14 ± 0.08
Significance	***	***	NS	NS	NS
MSD	0.001	0.054	0.741	0.098	2.464

Significance level: *** *p* ≤ 0.001, NS (no significance). NOTE: Numbers in bold represent the maximum values and the underlined numbers represent the minimum values.

**Table 7:** Bioactive compounds in bean varieties produced and consumed in the State of Oaxaca.

Varieties of Bean	Total Phenols (mg Gallic Acid/g)	Flavonoids (mg CE/g)	Anthocyanins (mg/g extract)
Blanco Alubia (119)	81.6 ± 22.5	0.6 ± 0.4	**2.33 ± 0.06**
Biche (123)	58.9 ± 24.6	0.7 ± 0.5	1.50 ± 0.06
Blanco Michigan (124)	98.7 ± 14.6	1.7 ± 0.1	2.03 ± 0.1
Peruano (126)	**109.5 ± 31.0**	2.0 ± 0.1	1.80 ± 0.1
Sangre de Toro (127)	94.9 ± 12.0	4.2 ± 0.2	1.16 ± 0.2
Bayo (128)	74.3 ± 9.7	4.1 ± 0.0	0.44 ± 0.2
Negro Bola (129)	84.0 ± 34.5	4.3 ± 0.09	0.87 ± 0.05
Negro Michigan (134)	91.2 ± 23.2	3.6 ± 0.3	0.84 ± 0.1
Bayo Bola (135)	104.7 ± 21.8	0.8 ± 0.2	0.45 ± 0.04
Flor de Mayo (137)	77.6 ± 13.6	4.1 ± 0.1	0.39 ± 0.04
Pinto Americano (140)	84.9 ± 13.5	**7.4 ± 0.1**	1.26 ± 0.07
Significance	**	NS	***
MSD	47.6	12.1	0.3

Significance level: ** *p* ≤ 0.01, *** *p* ≤ 0.001, NS (no significance). Note: Numbers in bold represent the maximum values and the underlined numbers represent the minimum values.
